# Genome-Wide Acetylation Modification of H3K27ac in Bovine Rumen Cell Following Butyrate Exposure

**DOI:** 10.3390/biom13071137

**Published:** 2023-07-16

**Authors:** Xiaolong Kang, Chenglong Li, Shuli Liu, Ransom L. Baldwin, George E. Liu, Cong-Jun Li

**Affiliations:** 1 Animal Genomics and Improvement Laboratory, Henry A. Wallace Beltsville Agricultural Research Center, Agricultural Research Service, U.S. Department of Agriculture, Beltsville, MD 20705, USA; kangxl@nxu.edu.cn (X.K.);; 2 Key Laboratory of Ruminant Molecular and Cellular Breeding, College of Animal Science and Technology, Ningxia University, Yinchuan 750021, China

**Keywords:** butyrate, H3K27ac, acetylation, cattle, rumen, super-enhancer

## Abstract

Butyrate contributes epigenetically to the changes in cellular function and tissue development of the rumen in ruminant animals, which might be achieved by its genetic or epigenetic regulation of gene expression. To explore the role of butyrate on bovine rumen epithelial function and development, this study characterized genome-wide H3K27ac modification changes and super-enhancer profiles in rumen epithelial primary cells (REPC) induced with butyrate by ChIP-seq, and analyzed its effects on gene expression and functional pathways by integrating RNA-seq data. The results showed that genome-wide acetylation modification was observed in the REPC with 94,675 and 48,688 peaks in the butyrate treatment and control group, respectively. A total of 9750 and 5020 genes with increased modification (H3K27ac-gain) and decreased modification (H3K27ac-loss) were detected in the treatment group. The super-enhancer associated genes in the butyrate-induction group were involved in the AMPK signaling pathway, MAPK signaling pathway, and ECM-receptor interaction. Finally, the up-regulated genes (*PLCG1*, *CLEC3B*, *IGSF23*, *OTOP3*, *ADTRP*) with H3K27ac gain modification by butyrate were involved in cholesterol metabolism, lysosome, cell adhesion molecules, and the PI3K-Akt signaling pathway. Butyrate treatment has the role of genome-wide H3K27ac acetylation on bovine REPC, and affects the changes in gene expression. The effect of butyrate on gene expression correlates with the acetylation of the H3K27ac level. Identifying genome-wide acetylation modifications and expressed genes of butyrate in bovine REPC cells will expand the understanding of the biological role of butyrate and its acetylation.

## 1. Introduction

The digestive tract greatly influences the maintenance of energy requirements in cattle due to its crucial role in digesting and absorbing nutrients. The rumen is an essential organ in the digestive tract for feed efficiency, with relatively high metabolic activities. The digestion of solid-feeding materials generates short-chain fatty acids (SCFAs) through fermentation via the action of anaerobic microorganisms in the rumen, which also affects the rumen’s physiological functions. The SCFAs are essential energy resources for ruminants and affect rumen epithelium integrity and renewal. At the same time, a healthy rumen also impacts SCFAs’ transport and absorption [1,2].

In ruminants, SCFAs constitute a significant energy source, contributing up to 70% of their energy requirements. Butyrate is also one of the SCFAs, plays a prominent role in multiple cellular functions, such as cell growth, differentiation, and apoptosis [3,4,5], and functions in various types of cells due to their capacity to modulate gene expression involved in cell function through modification of histone [6,7,8]. For farm animals, butyrate is reported to modulate the gut microbial metabolites [9] and the intestinal barrier [10,11], alleviate inflammation [12,13], and improve animal growth performance and meat quality [14,15]. All these might be achieved by butyrate-derived metabolic rewiring and genetic and epigenetic reprogramming, because butyrate could regulate gene expression changes by mediating multiple signal pathways [16,17,18] and epigenetic modification [7,19,20,21] at a bulk and single-cell level.

Butyrate is well known for its effects as a histone deacetylase inhibitor (HDACi), which can regulate gene expression. Gene expression involves a change in chromatin structure, which can be regulated by epigenetic mechanisms such as histone modification. Animal and human studies have revealed that the HDACi effect of butyrate makes it a significant inducer of the hyperacetylation of histone by inhibition of histone modification enzymes [22,23] and modification of the chromatin conformation [24], because acetylation of histone proteins has been associated with active transcription and weakens DNA-histone interaction to enhance chromatin accessibility [25]. Histone 3 lysine 27 acetylation (H3K27ac) is one of the histone marks that is highly enriched in active enhancers and promoters [26], which are accessible to transcription factors and other transcriptional complexes for regulating the transcription of genes [27]. Binding transcription factors to enhancers is the first step in gene expression. Super enhancers (SEs) are regions of the mammalian genome that contain multiple enhancers; super-enhancers exhibit higher densities of transcription factors and higher densities of transcriptional activation-related histone modifications (H3K27ac, etc.) than typical enhancers, and are frequently associated with the essential control of cell state and cell differentiation-specific genes, make a more substantial contribution to gene expression, and are mainly responsible for regulating the expression of genes that determine cell identity [28,29]. The other studies also indicated that ubiquitous chromatin opening elements marked by H3K27ac and DNase I can support the stable expression of nearby genes [30].

The cell is the primary living organism unit with specific information about the whole organism. However, many of the studies of the biological effects of butyrate in ruminants were based on the MDBK cell line, which was the only available cell line from cows. Complete characterization of the established primary cell culture to obtain the crucial genotyping information is inevitably required for an in vitro cell experimental system.

Primary cell culture has been extensively used in basic biological research as a model system. Those primary cell culture model systems retain great promise for useful information on many aspects of biology. Using primary cells also allows the researchers to avoid complications in animal models, such as availability, cost, and ethics. Therefore, we established a stable rumen epithelial primary cell (REPC) culture from a two-week-old Holstein bull calf rumen epithelial tissue as a critical and high-value tool to study the development of the rumen [7].

Regarding the HDACi effect mediated by butyrate, we profiled the genome-wide transcriptome change [17], CTCF-binding regions [19], and functional annotation of chromatin states under the butyrate treatment in our previous studies. However, it remains unknown how the H3K27ac mark changes under this treatment in rumen epithelial primary cells (REPC) and how H3K27ac histone acetylome changes contribute to the phenotype response in REPC under the butyrate treatment. Histone acetylome studies using the H3K27ac mark have successfully identified chromatin regulation changes under different conditions. To improve our understanding of response changes of REPC under butyrate treatment, in this study we employ chromatin immunoprecipitation followed by a sequencing (ChIP-seq) approach to characterize the H3K27ac binding landscape and integrate it with the transcriptome data to detect the relationship of H3K27ac and gene expression, and to better understand the gene regulatory mechanism of REPC under the butyrate induced.

## 2. Materials and Methods

### 2.1. Rumen Epithelial Primary Cells and Butyrate Treatment

Sample collections, primary rumen epithelial cells and butyrate treatment: in the current study, all animal procedures were conducted under the approval of the Beltsville Agricultural Research Center (BARC) Institutional Animal Care Protocol Number 15-008**.** Rumen primary epithelial cells were isolated from a two-week-old Holstein bull calf fed with milk replacer only. The rumen epithelial cell isolation and culture methods were reported previously [7]. Briefly, rumen epithelial tissue was collected from a two-week-old Holstein bull calf fed with milk replacer only. The epithelial layer of the rumen tissue was separated manually from the muscular layer. After rinsing in tap water to remove residual feed particles, samples were further rinsed in ice-cold saline. The tissue was then added to a 50 mL digestion solution (2% trypsin and 1.15 mmol CaCl2 in phosphate-buffered saline) and was then incubated in a 37 °C incubator for 15 min. Rumen epithelial fragments generally underwent 5–6 digestion cycles with fresh trypsin solution. Only the third, fourth, and fifth rounds of digestion were collected and combined for culture. Collected rumen epithelial cells were plated in a 25 cm plate at 1 million cells/dish density in DMEM with antibiotic-antimycotic and 5% fetal bovine serum (DMEM-FBS). The cell media were removed and replaced with fresh DMEM-FBS after 24 h in culture. Subsequently, cell media was changed every 48 h until the cells achieved confluence (4–7 days). Trypsinization removed cells from the dish; they were then quantified, reseeded for treatment, or frozen in liquid nitrogen for future culture.

For the current experiment, REPCs were cultured to 60% confluence before the experiment. Three replicate flasks of cells for both treatment and control groups (6 samples) were prepared for the final ChIP experiment with 5 × 10^7^ cells from each sample.

Butyrate treatment of cell culture: butyrate induces cell cycle arrest at the G1/S boundary. It also induces apoptosis at high concentrations [31]. The inherent VFA dependence of ruminant cells was exploited to increase sensitivity for studying butyrate’s role in gene response and biochemical regulatory pathways. Based on our previous experiment with bovine MDBK cells and REPCs [7], treatment of 5 mM butyrate in vitro for 24 h can induce significant changes in histone acetylation level and transcription activities without inducing substantial apoptosis and cell-cycle arrest. REPC has a similar reaction to the butyrate treatment. Therefore, the final dosage of 5 mM butyrate was added to the culture medium for 24 h for the butyrate treatment of cells.

### 2.2. Chromatin Immunoprecipitation and Sequencing

Samples of REPC (butyrate-induced (H3K27ac-BT/REPC-BT) and normal control (H3K27ac-CO/REPC-CO)) were prepared as in a previous study [7]. The reliability of the antibody was validated using Western blot and immune precipitation with cell lysates from butyrate-treated and untreated bovine cells. The antibody reacted strongly to butyrate-induced accumulation of acetyl-H3K27 with a single band in Western blot. The antibody also worked well in immunoprecipitation against acetyl-H3K27 in bovine MDBK cells, as we previously reported [32].

This work is a component of the functional annotation of cattle genomes, a coordinated international effort, the Functional Annotation of Animal Genomes (FAANG) project. The FAANG project closely follows the human ENCODE project. ChIP-sequencing experiments followed ENCODE guidelines with deep sequencing. Briefly, DNA recovered from a conventional ChIP procedure was quantified, and the DNA fragment’s size (200–500 bp) and integrity were verified using Agilent Bioanalyzer 2100. Three DNA samples from either control or butyrate-treated cells were pooled. ChIP-seq libraries (H3K27ac) of two groups (pooled) were constructed following the manufacturer’s protocol with end repair, adaptor ligation, and size selection. The libraries were validated and sequenced at 75 bp per sequence, read at a depth of approximately 40 million sequences per sample (mean ± SD = 44 ± 2.9 million per sample) [20]. After obtaining raw data, FastQC (v.0.11.9) was used to check read quality [33], and Trim Galore (0.4.0, default parameter) [34] was applied to trim low-quality (MAPQ < 25) reads. SAMtools (v.1.9) [35] was used to filter reads, and only uniquely mapped reads were kept for peak calling. Clean reads were mapped against the cattle reference genome (ARS-UCD1.2), using the BWA package (v.0.7.17, allowing two mismatches) [36]. Peaks were called using MACS2 (v.2.2.7.1) [37]. The H3K27ac data were normalized with control IgG, utilizing a package of deepTools2 (v3.5.1) [38]; reads coverage and enrichment profiles were analyzed by deepTools2(v3.5.1). The annotation of peak enrichment of H3K27ac was performed using ChIPpeakAnno (v3.0.0) [39] and ChIPseeker (v1.26.2) [40]. IGV (v.2.14) was used to visualize the genomic distribution of peaks with Bigwig files.

### 2.3. Differential Enrichment Regions and Super-Enhancer Identification

The differential peaks of H3K27ac were detected by MACS2 -bdgdiff (|log10 likelihood ratiol| > 3) and visualized by ChIPseeker (v1.26.2). ROSE (v.1.3.1, ROSE_main.py,-t 2500, ROSE_main.py and ROSE_geneMapper.py, searchwindow = default) was used to detect the super-enhancer [29]. ROSE stitches together peak regions less than 12.5 kb apart, then determines the number of tags (normalized alignment reads in the BED file) for each stitched region and ranks these regions by tag numbers; the top 5% of regions are defined as super-enhanced regions. R package clusterProfiler [41] and enrichplot were adopted to analyze gene enrichment and visualize gene set enrichment analysis (GSEA) results, respectively.

### 2.4. RNA-Seq Analysis

RNA-seq data were downloaded from a previous study (BioProject ID: PRJNA531209, three replicates for each group). HISAT2 (v2.2.1) [42] was used to map reads to the bovine reference genome (ARS-UCD1.2); StringTie (v2.1.5) [43] and DEseq2 [44] were used separately to count reads (transcripts per million, TPM) and detect differentially expressed genes (DEGs) (|log2FC| ≥ 1, false discovery rate (FDR) < 0.05).

### 2.5. Functional Enrichment and Pathway Analysis

ShinyGO (v.0.75) software was used to explore enrichment in Gene Ontology (GO) categories and the KEGG functional pathway [45]; multiple testing correction (Benjamini–Hochberg) was applied, and a process with FDR < 0.05 was considered significant.

### 2.6. Protein-Protein Interaction Analysis

The protein–protein interaction analysis was conducted for genes of interest on STRING (https://string-db.org/, v11.5, accessed on 15 January 2023), and the core genes were identified using the plugin CytoHubba in Cytoscape (MMC algorithm).

## 3. Results

### 3.1. Genome-Wide Changes of H3K27ac Profiles in Butyrate-Induced REPC

**Figure 1:**
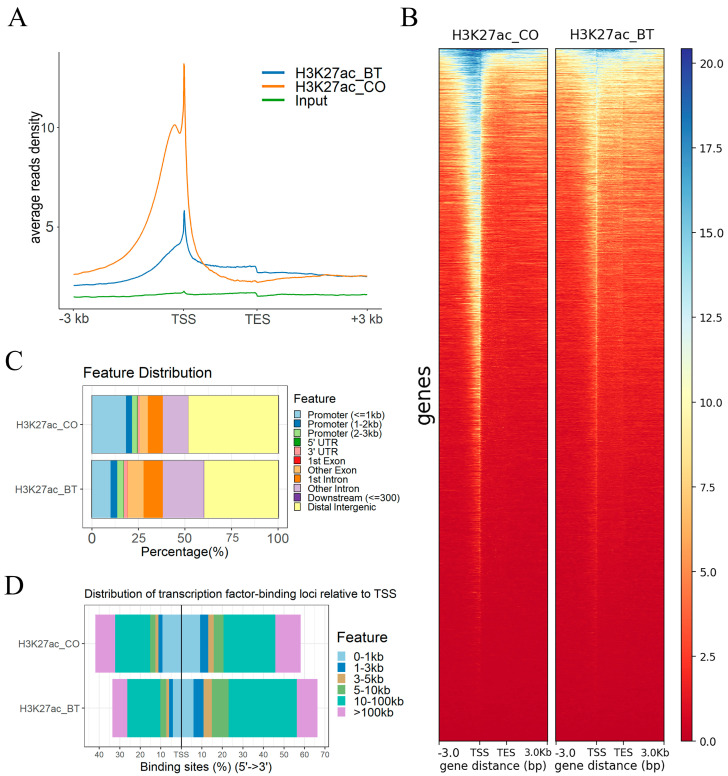
Genome-wide profiling of H3K27ac acetylation in REPC. (**A**) Characterization of the H3K27ac peak distribution pattern in butyrate-treated and control group. The x-axis represents different regions of a peak on the genome, and the y-axis represents the normalized average read density. (**B**) Heatmap profile of H3K27ac peaks close to the transcription start sites (TSS), containing a ±3 kb region for each group. Blue color intensity reflects the level of peak enrichment. (**C**) Annotation of H3K27ac binding peaks in butyrate-treated and control group. (**D**) Distribution of H3K27ac binding peak relative to TSS in butyrate-treated and control group. BT: butyrate. CO: control.

ChIP-seq was used to analyze the genomic features of H3K27ac to identify the association of histone modification (acetylation) and butyrate treatment in bovine REPC. A total of 94,675 and 48,688 peaks of H3K27ac were detected in the butyrate and control groups, respectively (Table S1, Figure S1). The H3K27ac peak signals enrich clearly at upstream regions (±3 kb) from the transcription start site (TSS), and there are weak peak signals around the transcription end site (TES) in butyrate-induced and control groups (Figure 1A,B). It is noteworthy that butyrate treatment induced more peaks but with weaker intensity compared to the control, which means extensive but low levels of acetylated histones were observed in the butyrate-induction group (Figure 1A,B). While these results are unexpected, previous reports have found that many genomic regions close to transcription start sites were deacetylated after butyrate exposure, and butyrate treatment also induced an apparent decrease in the intensity of the peaks. This unexpected result might also correlate with the down-regulation of essential transcript factor *TAF1* (a TATA-box binding protein-associated factor of the RNA polymerase II transcription initiation factor complex TFIID) possessing histone acetyltransferase (HAT) activity in this butyrate-inducted cell (Table S2). The peak annotation of H3K27ac showed that most peaks were enriched mainly in distal intergenic regions, promoters (<=1kb), and introns (Figure 1C, Figure S2). Regarding peak distance relative to TSS, most peaks are located at 10–100 kb and >100kb of TSS (Figure 1D, Figure S2).

### 3.2. H3K27ac Modified Regions That Regulate Gene Profiles in Butyrate-Induced REPC

To investigate whether the levels of the H3K27ac marker changed significantly after butyrate treatment, differential enrichment of H3K27ac peaks was identified in REPC-BT and REPC-CO groups using MACS2, while the associated genes annotated to these regions were defined as differentially H3K27ac-modified genes (DHGs). A total of 53,128 differential enriched peaks were proximal to 10,356 genes between the two groups (Table S3). Butyrate induced 9746 DHGs with increased modification (H3K27ac-gain) and 5018 DHGs with decreased modification (H3K27ac-loss) in REPC-BT. Despite limited in vitro culture, variations in the H3K27ac modification were present in rumen epithelial cells under butyrate induction.

**Figure 2:**
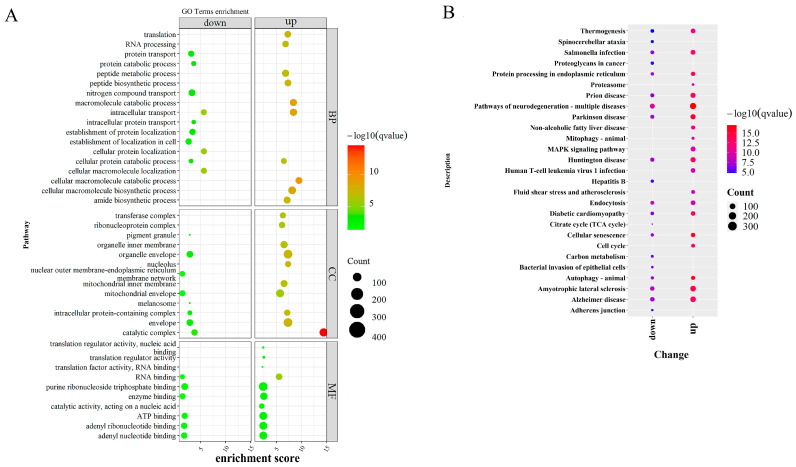
Functional enrichment and pathway analysis of genes modified. (**A**) GO enrichment analysis of DHG showing top GO terms for biological processes, cellular components, and molecular function. The color represents the magnitude of the −log10 (FDR) value. The size of the bubbles indicates the number of genes. (**B**) KEGG functional enrichment analysis of DHG. The color represents the magnitude of the −log10 (FDR) value. The size of the bubbles indicates the number of genes.

We further focused on the GO functions of DHGs modified by the differentially enriched peaks to understand the potential function of these genes. The results showed that modified genes with H3K27ac-gain regions were associated with biological processes, such as the amide biosynthetic, mitotic cell cycle, and mRNA metabolic processes. Meanwhile, genes with H3K27ac-loss regions were mainly related to protein synthesis and catabolism, including nitrogen compound transport, protein transport, the establishment of protein localization, and the protein catabolic process (Figure 2A). For KEGG pathways, modified genes with H3K27ac-gain regions were involved in the proteasome, mitophagy, and MAPK (mitogen-activated protein kinase) signaling pathway, yet genes with H3K27ac-loss regions were enriched primarily on the carbon metabolism citrate cycle (TCA cycle) (Figure 2B).

### 3.3. Genome-Wide H3K27ac-Based Super-Enhancer Detection between Groups

**Figure 3:**
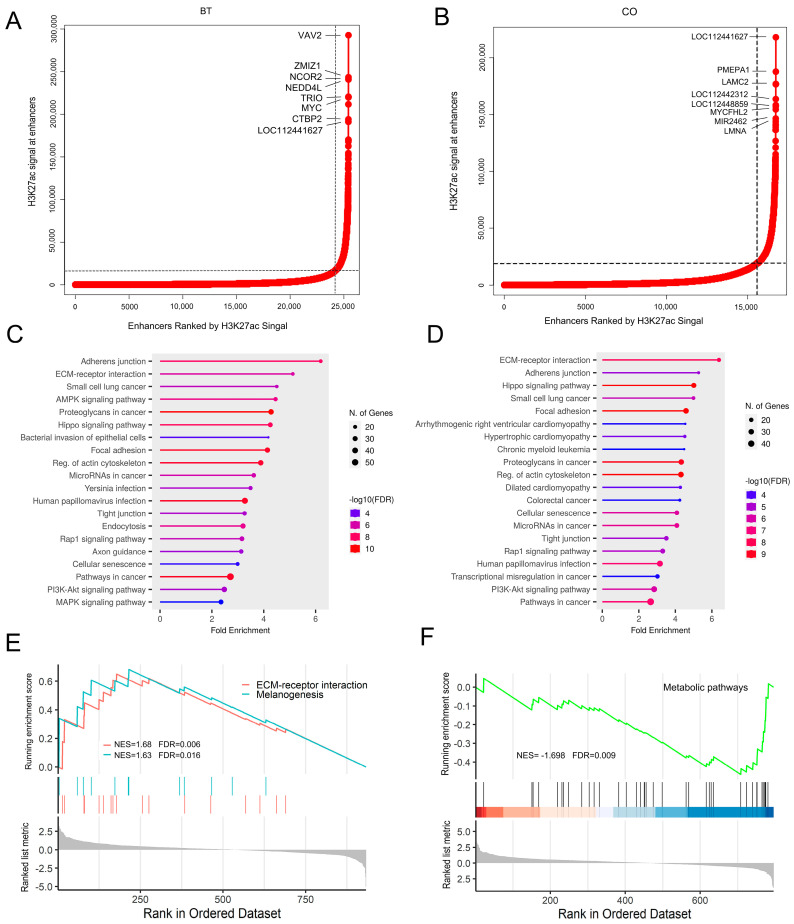
Identification of H3K27ac-based super-enhancers in REPC**.** (**A**,**B**). Ranking of H3K27ac ChIP-seq signal between butyrate-induced and control groups. (**C**,**D**) KEGG enrichment analysis of super-enhancer-associated genes. The color represents the magnitude of the -log10 (FDR) value. The size of the bubbles indicates the number of genes. (**E**,**F**) GSEA results for super-enhancer-associated genes. Normalized enrichment score (NES) and false discovery rate (FDR) are listed for the analyses. BT: butyrate. CO: control.

Ranking ChIP-Seq signals could reveal a super-enhancer (SE) that displays increased enrichment of enhancer-associated histone mark (H3K27ac) in a cell-specific and status-specific manner. In this study, H3K27ac-based SEs were identified between REPC-BT and REPC-CO groups to determine super-enhancer landscapes and clarify the dynamic of epigenetic status in butyrate-induced REPC. A total of 1186 and 1118 SEs were detected between the treatment and control groups (Figure 3A,B, Table S4). The genes with the most significant SE enrichment signals in the treatment group included *VAV2*, *ZMIZ1*, *NCOR2*, etc. Super-enhancers promote strong expression of cell recognition-related genes; some SE-related genes that are present in both BT and CO groups may be associated with cell identity, such as *MYC* and *NEDD4L*, which are associated with the proliferation and differentiation of epithelial cells [46,47]. The KEGG pathway enrichment analysis showed that genes associated with SEs were exclusively involved in the AMPK signaling pathway, MAPK signaling pathway, and endocytosis in the REPC-BT group (Figure 3C,D). Furthermore, gene enrichment analysis revealed that genes related to metabolic pathways were down-regulated in the control group. In contrast, genes associated with ECM-receptor interaction and melanogenesis were up-regulated in the butyrate-induced group (Figure 3E,F). ECM-receptor interactions primarily mediate interactions with cell adhesion receptors to regulate the adhesion, motility, and growth of epithelial cells [48,49].

### 3.4. Integrative Analysis of Butyrate-Associated Transcriptome and H3K27ac Modification

**Figure 4:**
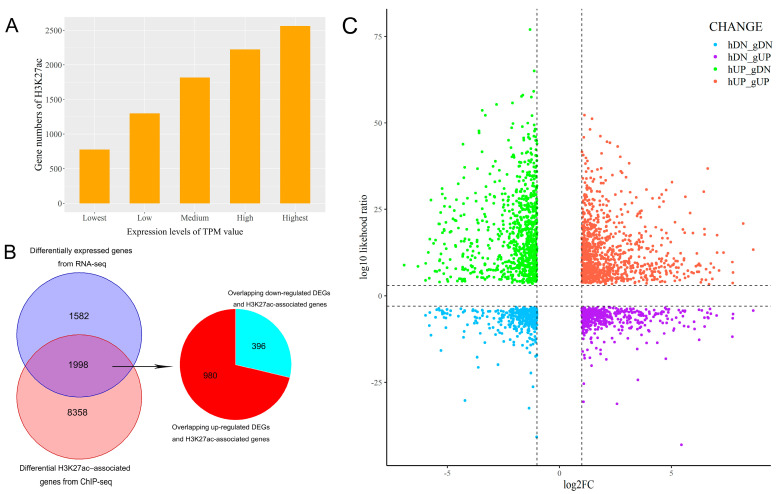
The effects of H3K27ac on the expression of its marked genes. (**A**) Changes in gene numbers marked by H3K27ac among five groups (lowest, low, medium, high, and highest) based on their expression levels. (**B**) Venn diagram showing the overlap of 3580 DEGs from RNA-seq data and 10,356 differential H3K27ac-associated genes from ChIP-seq data. A set of 1998 genes in the overlapping regions were identified, including 980 up-regulated DEGs with increased H3K27ac levels and 396 down-regulated DEGs with decreased H3K27ac levels. (**C**) Plots of log2FoldChanges in gene expression and log10 likelihood ratio in H3K27ac enrichment. Genes with significant increases or decreases in H3K27ac and gene expression are highlighted in red and blue, respectively.

Since histone acetylation is associated with transcriptional activation, we investigated the relationships between butyrate-associated gene expression and H3K27ac profiles in the REPC under butyrate treatment. First, after all expressed genes and DEGs were detected between REPC-BT and REPC-CO groups (Figure S3, Table S2), all expressed genes were divided into five groups according to their expression levels (TPM), in descending order. Each group contained the same number of genes (2915 genes), and the highest expressed gene group was labeled as “highest”; the following groups were labeled as “high”, “medium”, “low”, and “lowest”, in that order. We compared the number of H3K27ac-modified genes in the five groups and found that the number of H3K27ac-modified genes was positively correlated with their expression levels (Figure 4A).

The integrative analysis of ChIP-seq and RNA-seq data showed that 1,998 identified DEGs (for more details see Supplementary File Table S5) were overlapped with DHGs, of which 980 up-DEGs (49%) possessed the H3K27ac-gain signal, and 396 down-DEGs (20%) owned the H3K27ac-loss signal (Figure 4B,C, Figure S4), indicating that there is a correlation between changes in gene expression and H3K27ac modification in rumen epithelial cells which were butyrate-induced. In detail, the increase in the expression levels of the H3K27ac-gain region containing genes (*PLCG1*, *CLEC3B*, *IGSF23*, *OTOP3*, *ADTRP*, *ZAP70*, etc.) was significantly associated with enhanced H3K27ac signals. Similarly, genes (*MRTO4*, *CKS2*, *POLE*, etc.) were down-regulated in expression, while the peak enrichment was decreased (Table 1, Figure S5). We also counted the changes in the expression of genes related to the biological functions of rumen epithelial cells (Table S6).

A function analysis of these DEGs overlapped with DHGs was conducted to further understand their biological roles with the KEGG database (FDR < 0.01). The results showed that DHG genes of H3K27ac-gain and up-regulated DEG expression (hUP-gUP) were primarily involved in cholesterol metabolism, lysosome, cell adhesion molecules, and the PI3K-Akt signaling pathway. In contrast, the DHG genes of H3K27ac-loss and down-regulated DEG expression (hDN-gDN) were related to cell proliferation (such as ribosome biogenesis in eukaryotes, pyrimidine metabolism, the cell cycle, and cellular senescence) and DNA replication and repair (the Fanconi anemia pathway and P53 signaling pathway) (Figure S6).

**Figure 5:**
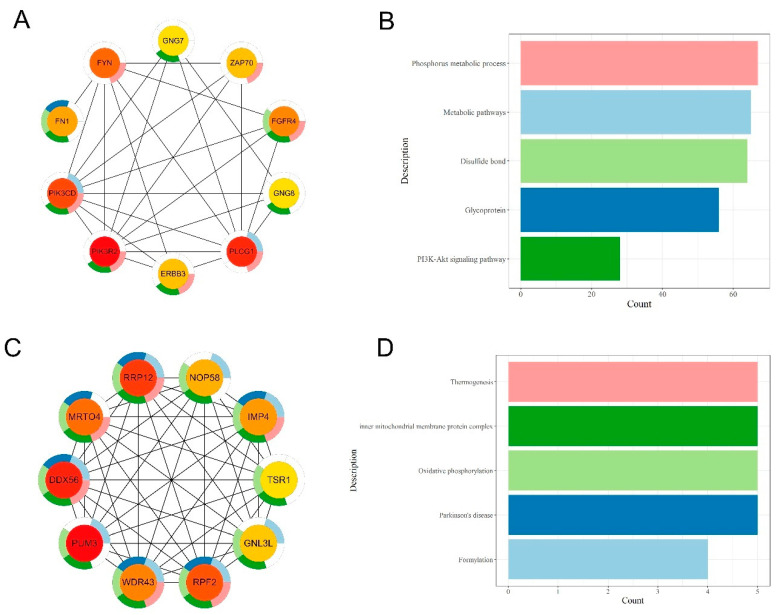
PPI analysis of hUP-gUP and hDN-gDN genes. (**A**,**B**) hUP-gUP core genes in the PPI network and their enrichment pathways. The color of the outer circle of the gene represents the pathway. (**C**,**D**) hDN-gDN core genes in the PPI network and their enrichment pathways. The color of the outer circle of the gene represents the pathway.

**Figure 6:**
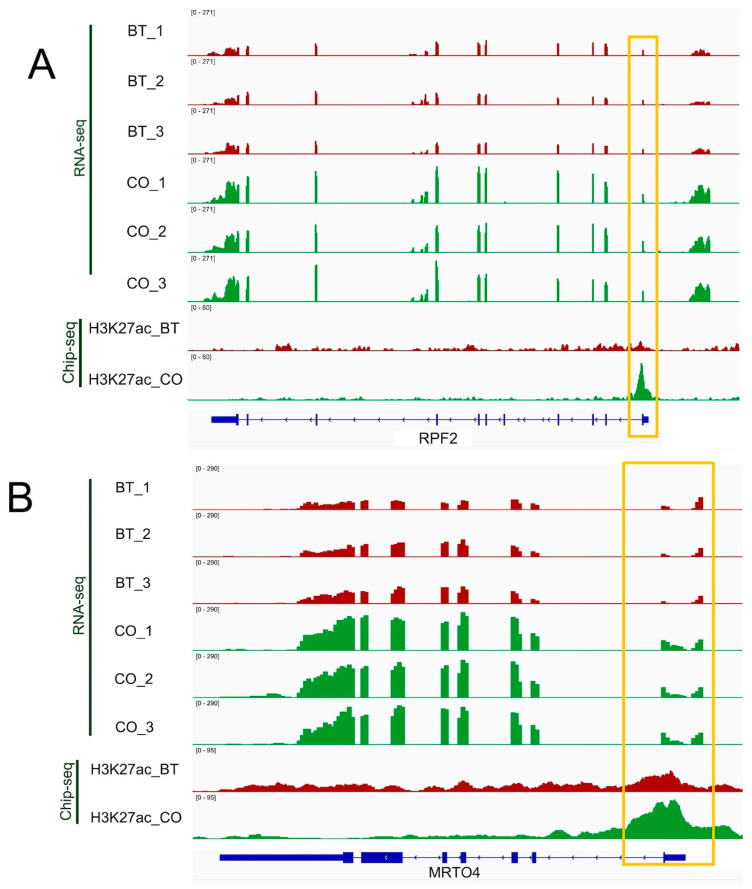
Genomic tracks of H3K27ac ChIP-seq and RNA-seq data of genes in butyrate-treated and control groups. (**A**) RPF2 gene shows a sharp pattern, significant decreases in H3K27ac, and decreased gene expression. (**B**) MRTO4 gene shows a dispersed pattern, with substantial reductions in both H3K27ac and gene expression. X-axis: genomic location of the gene of interest. Y-axis: normalized read counts. Data from the butyrate-treated group are in red, whereas data from the control group are in green.

We then performed PPI analysis and identified 20 core genes. Among them, the core genes of hUP-gUP were associated with metabolic pathways, glycoprotein, the disulfide bond, the PI3K-Akt signaling pathway, and the phosphorus metabolic process (Figure 5A,B). The core genes of hDN-gDN are mainly involved in thermogenesis, the inner mitochondrial membrane protein complex, oxidative phosphorylation, Parkinson’s disease, and formylation (Figure 5C,D). As shown in IGV (Figure 6), for the core genes *RPF2* and *PIK3R2*, their gene expression changes, accompanied by a peak enrichment signal of H3K27 acetylation. The H3K27ac mark shows different patterns (sharp vs. dispersed) in these two genes. This phenomenon was also presented in our earlier report, with bovine MDBK cells [32]. The H3K27ac binding pattern might be related to gene structure, such as the gene size and sizes of the intron, exon, etc.).

## 4. Discussion

Short-chain fatty acids (SCFAs) are nutrients especially critical to ruminants, formed during the microbial fermentation of dietary fiber. Whereas acetate and propionate are major components of SCFAs that provide energy in ruminant metabolism, butyrate appears to be not only involved in energy metabolism but also serves as an inhibitor of HDACs, which are critical epigenetic regulators [6,31,50,51]. Through its HDACi effects, butyrate induces an accumulation of histone acetylation and regulates genetic activities. Previous studies showed that H3K27 acetylation is vital in regulating gene expression under a butyrate induction [52,53]. However, it remains unknown what effect the butyrate induction has on the bovine epithelial primary cell. Here, we sought to investigate the biological effects of butyrate induction in bovine rumen primary epithelial cells and to understand the epigenetic modifying role and possible mechanisms of molecular changes in cells mediated by butyrate treatment.

In our earlier report, analysis of the transcriptome indicated that butyrate could induce differentiation, development, and growth of cattle epithelial tissue by modifying the expression of several genes in REPC [7]. The role of butyrate in the development of ruminal epithelium and its effect on isolated epithelial cells has been receiving research attention recently, and it has been established that butyrate is essential as a substrate of cell metabolism and as a regulator of gene activities.

In this study, we detected the genome-wide changes of histone acetylation in REPC cells after butyrate treatment, using a typical histone modification marker (H3K27ac). The results displayed more quantitative peaks in TSS proximal regions in a butyrate-treated group than in the control group, indicating that butyrate induction has a histone modification on the promoter region of a gene by mediating a TSS-centered H3K27ac reduction. In contrast to the global increases in histone acetylation, the enrichment signals of H3K27 acetylation were weaker at many genomic regions close to TSS after butyrate exposure, which is similar to other studies of hepatocarcinoma HepG2 cells and bovine mammary epithelial cells treated by butyrate [53,54].

The histone acetylation depends on histone deacetylase (HDAC) and histone acetyltransferase (HAT). Theoretically, butyrate could reactivate epigenetically silenced genes by increasing genomic histone acetylation; in contrast, there was a deacetylation modification in the treatment group in the study, consistent with other reports [53,54]. This paradox could be associated with the pleiotropic effects of butyrate, which can affect non-histone targets (e.g., transcription factors) [55], suggesting the H3K27 acetylation modification in bovine REPC by butyrate might be compromised by other essential transcription factors or DNA-binding proteins at their enrichment sites. HAT *TAF1*, a TATA-box binding protein-associated factor (TAF) of the RNA polymerase II transcription initiation factor complex TFIID, is integral to the regulation of eukaryotic transcription initiation and plays a critical role in the preinitiation complex (PIC) assembly [56,57,58]. The HAT activity of *TAF1* is essential for transcription from a subset of genes and G1 progression in mammalian cells [59]. The effects of G1 cell cycle arrest [60] and down-regulation of gene expression [61] caused by disruption of *TAF1* HAT activity are similar to those observed during butyrate treatment [53], which is consistent with the down-regulation of *TAF1* in the butyrate-induced group in this study.

Moreover, a major contradiction is how the increase in global histone acetylation level induced by butyrate can correlate with the decreased intensity of our ChIP-seq analysis of REPC. We also showed substantial gene reductions in H3K27ac and gene expression induced by butyrate, in the case of certain genes. The acetylation of histones results from the net activity of HAT and HDAC. Butyrate can cause oxidative stress in treated cells while promoting histone acetylation modifications [62,63]. However, some genes express via the recruitment of HDAC under oxidative stress conditions [51]. In a word, we speculate a direct or indirect decrease in HAT activity and an increase in HDAC during butyrate exposure in bovine rumen cells. This may also suggest that the presumed mechanisms of butyrate HDACi activity need to be tested more.

GO enrichment analysis of DHG showed that the DHG with H3K27ac-gain modification was mainly involved in translation and protein synthesis, while DHGs with H3K27ac-loss modification were associated with protein catabolism. Protein synthesis is affected by cell proliferation and the cell cycle. Butyrate is involved in bovine rumen development and cell proliferation, which can promote rumen epithelial maturation, so protein synthesis activity was speculated to be related to butyrate stimulation. We also identified KEGG pathways present only in DHGs of increased modification, such as Mitophagy and the MAPK signaling pathway. Studies reported that butyrate could protect the gastrointestinal barrier by providing energy to the gastrointestinal epithelium, histone deacetylation, and immunomodulation [64,65], because after butyrate treatment, Mitophagy activation via AMPK could alleviate oxidative stress, intestinal epithelial cell barrier damage, and mitochondrial damage [66]; butyrate protects β-cells from damage via the p38/MAPK signaling pathway, thereby preventing rumen epithelial barrier dysfunction [67].

Super-enhancers (SE) are clusters of transcriptional enhancers that promote gene expression of their adjacent genes. SEs are cell-specific and typically characterized by high levels of H3K27ac acetylation [68]. In this study, butyrate treatment-associated SE genes were up-regulated in the treatment group’s ECM–receptor interaction and melanogenesis pathways. In contrast, in the control group, cell-specific associated SE genes were down-regulated in metabolic pathways, indicating SE-associated genes were associated with the biological functions of the cells under treatment-specific (REPC-BT) or cell-specific (REPC-CO) conditions. ECM–receptor interaction primarily regulates cell adhesion, migration, proliferation, and differentiation, and mediates interactions with cell adhesion receptors with essential roles in maintaining cell and tissue structure [49]. This pathway may be involved in signal transduction regulation of bovine rumen epithelial cell proliferation, consistent with previous results in other species and tissues [48,69]. Down-regulated expression of metabolic pathway-related genes in the control group indicated that the metabolic activity of REPC cells might be increased under butyrate treatment. Combined with the enrichment pathways MAPK, PI3K-Akt, and Hippo in the treatment group, the functional pathway of butyrate-associated SE genes suggested that butyrate-associated SEs may play vital roles by driving the expression of genes that affect cellular metabolic processes and the active status of bovine rumen epithelial primary cell, because supplementation with exogenous butyrate reduces apoptosis and promotes rumen growth [70], increases metabolism-related gene expression in rumen epithelial cells [71], and improves bovine gastrointestinal development and production performance [72].

Butyrate can alter the level of epigenetic modifications and chromatin state of rumen cells, leading to changes in gene expression, which are essential for rumen growth and development [20]. Here, we characterized the profile of H3K27ac in butyrate-induced REPC; the integrative analysis of acetylome and transcriptome showed that H3K27ac-gain was significantly enhanced, while associated gene expression was increased upon butyrate treatment, whereas H3K27ac-loss was associated with decreased gene expression, suggesting that histone modifications of H3K27ac-gain or -loss may be related to gene transcription. And those genes of hUP-gUP and hDN-gDN most likely represent the direct effect of H3K27ac differential enrichment on gene expression; for example, *ADTRP*, *CLEC3B*, and *ZAP70* in the hUP-gUP group, and *CKS2*, *NEIL3*, and *NCAPG2* in the hDN-gDN gene set. *CLEC3B* has an anti-proliferative function mediated by the mitogen-activated protein kinase (MAPK) pathway [73]. ADTRP may promote proliferation by up-regulating cell cycle genes such as *CCND1* and *CDK4*, inhibit apoptosis by down-regulating apoptosis genes such as *CASP7* and *PDCD2* [74], and regulate lipid metabolism mediated by *PPAR* [75]. *NEIL3* initiates base excision repair to maintain genomic stability [76], and *NCAPG2* is involved in mitosis, DNA repair, and histone regulation [77] and has a vital role in inhibiting cancer cell proliferation [78].

Essential proteins play a crucial role in cellular life activity and help us understand cell organization principles and their functions. The PPI analysis results of hDN-gDN and hUP-gUP genes showed that the core genes of hUP-gUP are mainly involved in metabolic pathways, such as the PI3K-AKT signaling pathway, which is consistent with our analysis of SE. The integrated network of the PI3K-AKT signaling pathway and metabolism maintains cellular homeostasis and drives the cell cycle in response to mitotic stimulation [79]. *PI3K*, an essential component of the PI3K-AKT pathway, is a crucial regulator of cell growth, the cell cycle, and protein synthesis, and *PIK3R2* (Phosphoinositide-3-Kinase Regulatory Subunit 2) is one of the significant regulatory subunits of *PI3K*. It was shown that histone H3 acetylation is associated with the transcriptional regulation of *PIK3R2*. The accumulation of *PIK3R2* acetylation on TSS correlates with its expression level. Inhibition of histone deacetylase by HDACi treatment can induce deacetylation of the *PIK3R2* gene, while up-regulation of *PIK3R2* expression can increase cell proliferation through the PI3K/AKT signaling pathway and reduce apoptosis [80,81]. The core genes of hDN-gDN are mainly involved in thermogenesis, oxidative phosphorylation, and formylation. Lysine-specific demethylase (*LSD1*) is a histone enzyme demethylase and an essential regulator of the thermogenesis factor. Studies have shown that butyrate can mediate thermogenesis through *LSD1* and that reduced expression of *LSD1* impairs the butyrate-mediated thermogenesis induction [82,83].

ChIP-seq is a mature and reliable method to study protein-DNA interaction and established an essential global profile of the genome-wide H3K27 acetylation in this study. But functional validation of ChIP-seq data is still very challenging. There are two primary validation methods (qPCR and knockdown analysis). While limited by the ChIP chromatin for the qPCR test in this study, using knockdown analysis to functional test some of the significant H3K27ac binding sites remains a primary method to functionally validate the ChIP-seq data. However, that is certainly out of the scope of this report.

## 5. Conclusions

In this study, we globally profiled the genome-wide H3K27 acetylation and investigated the changes in transcription and histone modifications (H3K27ac) after butyrate treatment in bovine rumen epithelial primary cells. Butyrate treatment in REPC cells affects genome-wide acetylation levels of H3K27 and gene expression. The effect of butyrate on gene expression closely correlates with its acetylation level at the upstream region of the gene. Functional pathways of differentially enriched H3K27ac-modified genes, super-enhancer associated genes, and differentially expressed genes with H3K27ac gain in the treated group differ from the control, while treatment with butyrate may further impact the function of bovine rumen primary epithelial cells and affect rumen development. Future work must focus on cell types and histone markers or transcription factors closely related to acetylation to explore the molecular regulation of bovine rumen physiological activity by butyrate at the genomic level.

## Figures and Tables

**Table 1:** The gene list and functional description of the top 10 hUP-gUP and hDN-gDN genes.

Gene ID	Gene Symbol	H3K27ac	DEGs	Function Description
ENSBTAG00000019533	ADTRP	UP	UP	a serine hydrolase enzyme
ENSBTAG00000018331	CLEC3B	UP	UP	a protein-coding gene; encodes tetranectin
ENSBTAG00000048075	IGSF23	UP	UP	Immunoglobulin superfamily member 23
ENSBTAG00000016229	KLF9	UP	UP	a glucocorticoid-inducible factor
ENSBTAG00000013499	LHX9	UP	UP	LIM/homeobox protein; involved in gonadal development
ENSBTAG00000040111	OTOP3	UP	UP	Otopetrin 3
ENSBTAG00000046544	SLC7A10	UP	UP	neutral amino acid transporter
ENSBTAG00000013176	SMOC2	UP	UP	secreted protein acidic
ENSBTAG00000006419	TNNT1	UP	UP	Troponin T, slow skeletal muscle
ENSBTAG00000005892	ZAP70	UP	UP	Tyrosine protein kinase
ENSBTAG00000000590	POLE	DOWN	DOWN	DNA polymerase
ENSBTAG00000001938	CKS2	DOWN	DOWN	CDC28 protein kinase regulatory subunit
ENSBTAG00000005269	CCNB2	DOWN	DOWN	cyclin B2
ENSBTAG00000005825	NEIL3	DOWN	DOWN	DNA glycosylase
ENSBTAG00000007749	TONSL	DOWN	DOWN	DNA repair protein
ENSBTAG00000007783	MYBL2	DOWN	DOWN	a member of the MYB family,regulates cell proliferation
ENSBTAG00000008758	KIF20A	DOWN	DOWN	kinesin family member 20A
ENSBTAG00000010766	OIP5	DOWN	DOWN	Opa interacting protein 5
ENSBTAG00000016131	NCAPG2	DOWN	DOWN	non-SMC condensin II complex subunit G2
ENSBTAG00000016619	MIS18A	DOWN	DOWN	MIS18 kinetochore protein A

## Data Availability

All RNA sequencing data were submitted to NCBI, SRA database (SUB3040669, BioProject ID: PRJNA658627). All ChIP-sequencing data were submitted to NCBI, SRA database (SUB8420017, BioProject ID: PRJNA672996).

## Supplementary Materials

The following supporting information can be downloaded at: https://www.mdpi.com/article/10.3390/biom13071137/s1, Figure S1: Distribution of H3K27ac peaks along with chromosomes in butyrate-treated (H3K27ac-BT) and control (H3K27ac-CO) groups. Figure S2: Venn diagram of H3K27ac binding site annotation in butyrate-treated and control groups. CO and BT represent the control and butyrate-treated group, respectively. Figure S3: Transcriptome profiles of control and butyrate-treated groups. (A) Samples correlation heatmap of two groups. (B) Volcano map of differentially expressed genes (|log2FC| ≥ 1, FDR < 0.05). (C) KEGG enrichment analysis of up-regulated DEGs in the butyrate-treated group. (D) KEGG enrichment analysis of down-regulated DEGs in the butyrate-treated group. The color represents the magnitude of the −log10 (FDR) value. The size of the bubbles indicates the number of genes. CO and BT represent the control and butyrate-treated group. Figure S4: Plots of log2fold changes in gene expression and log10 likelihood ratio in H3K27ac enrichment. (A) Volcano map of hUP_gDN and hUP_gUP genes. (B) Volcano map of hDN_gDN and hDN_gUP genes. Figure S5: Genomic tracks of genes with ChIP-seq and RNA-seq data in butyrate-treated and control groups. (A) PLCG1 gene, significant increases in both H3K27ac signal and gene expression. (B) PIK3R2 gene, significant increases in both H3K27ac signal and gene expression. X-axis: Genomic location of the gene of interest. Y-axis: Normalized read counts. Data from the butyrate-treated group are in red, whereas data from the control group are in green. Figure S6: KEGG enrichment analysis of DEGs with differential enriched H3K27ac levels. (A) KEGG enrichment analysis of hUP-gUP gene. (B) KEGG enrichment analysis of hDN-gDN gene. The color represents the magnitude of the −log10 (FDR) value. The size of the bubbles indicates the number of genes. Table S1: Annotated information of H3K27ac peak in butyrate-treated and control groups. Table S2: Differentially expressed genes (|log2FC| ≥ 1, FDR < 0.05) between butyrate-treated and control groups. Table S3: Annotated information on differential enriched H3K27ac peaks in the butyrate-treated group. Table S4: Ranking and annotation information of super-enhancers in the butyrate-treated and control groups. Table S5: Annotation information of hUP-gUP and hDN-gDN genes. Table S6: A list of genetic changes in genes associated with rumen development.

## Abbreviations

REPC	rumen epithelial primary cell
SCFAs	short-chain fatty acids
HDAC	histone deacetylase
HDACi	histone deacetylase inhibitor
H3K27ac	histone H3 lysine 27 acetylation
IGV	integrative genomics viewer
DEG	differentially expressed gene
GSEA	gene set enrichment analysis
PPI	protein–protein interaction
TSS	transcription start site
TES	transcription end site
SE	super-enhancer
HAT	histone acetyltransferase
DHG	differentially H3K27ac-modified gene
TPM	transcripts per million
hUP-gUP	H3K27ac-gain and up-regulated DEG expression
hDN-gDN	H3K27ac-loss and down-regulated DEG expression
MAPK	mitogen-activated protein kinase
