# Supplementary figures and images for: Genome-Wide Acetylation Modification of H3K27ac in Bovine Rumen Cell Following Butyrate Exposure
Source: Biomolecules. 2023 Jul 16;13(7):1137. doi: 10.3390/biom13071137 (PMC10377523; doi:10.3390/biom13071137)

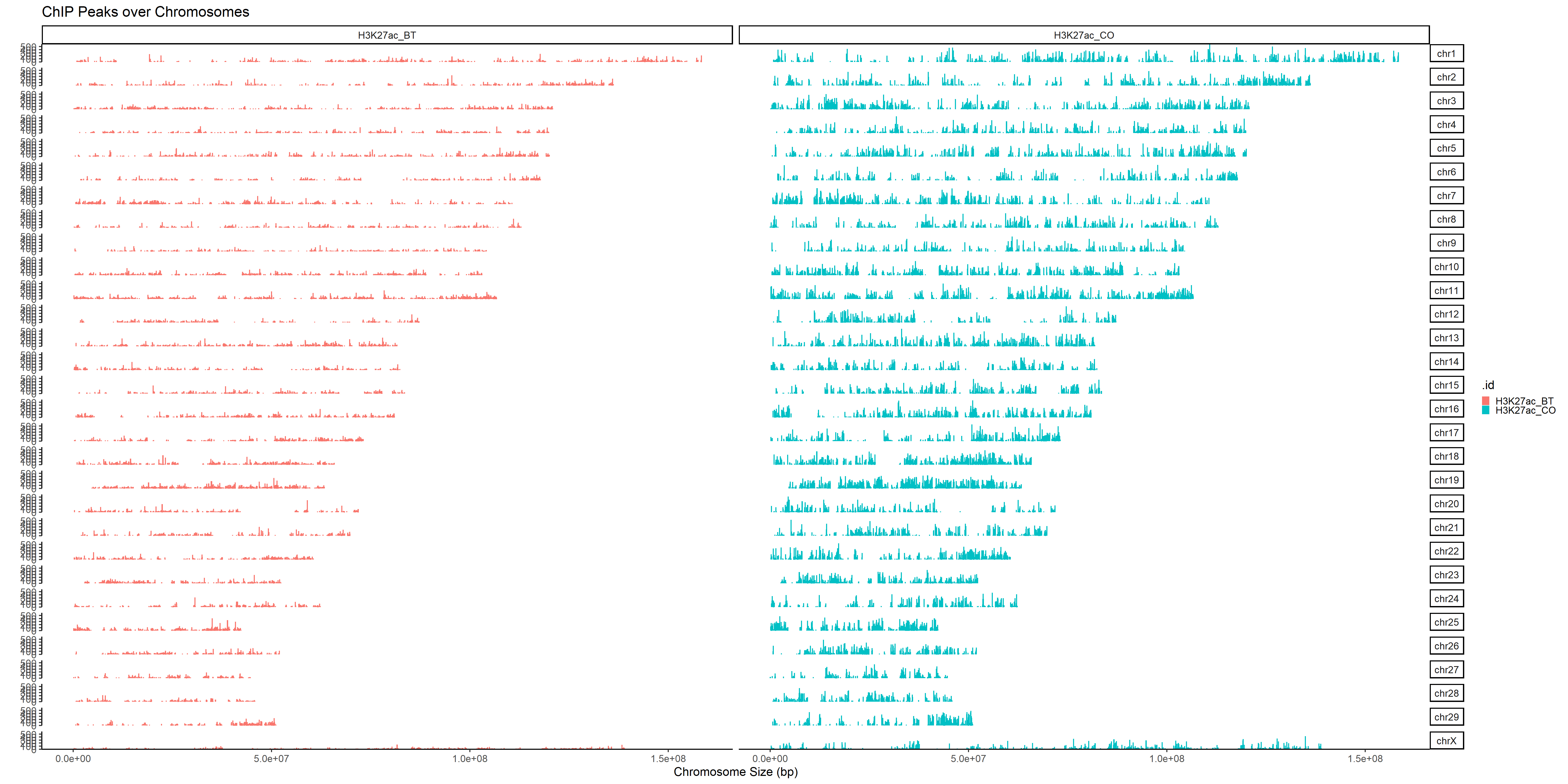

Supplement: Supplementary file 1 [file biomolecules-13-01137-s001.zip › Supplementary Figure/Figure S1.jpg]

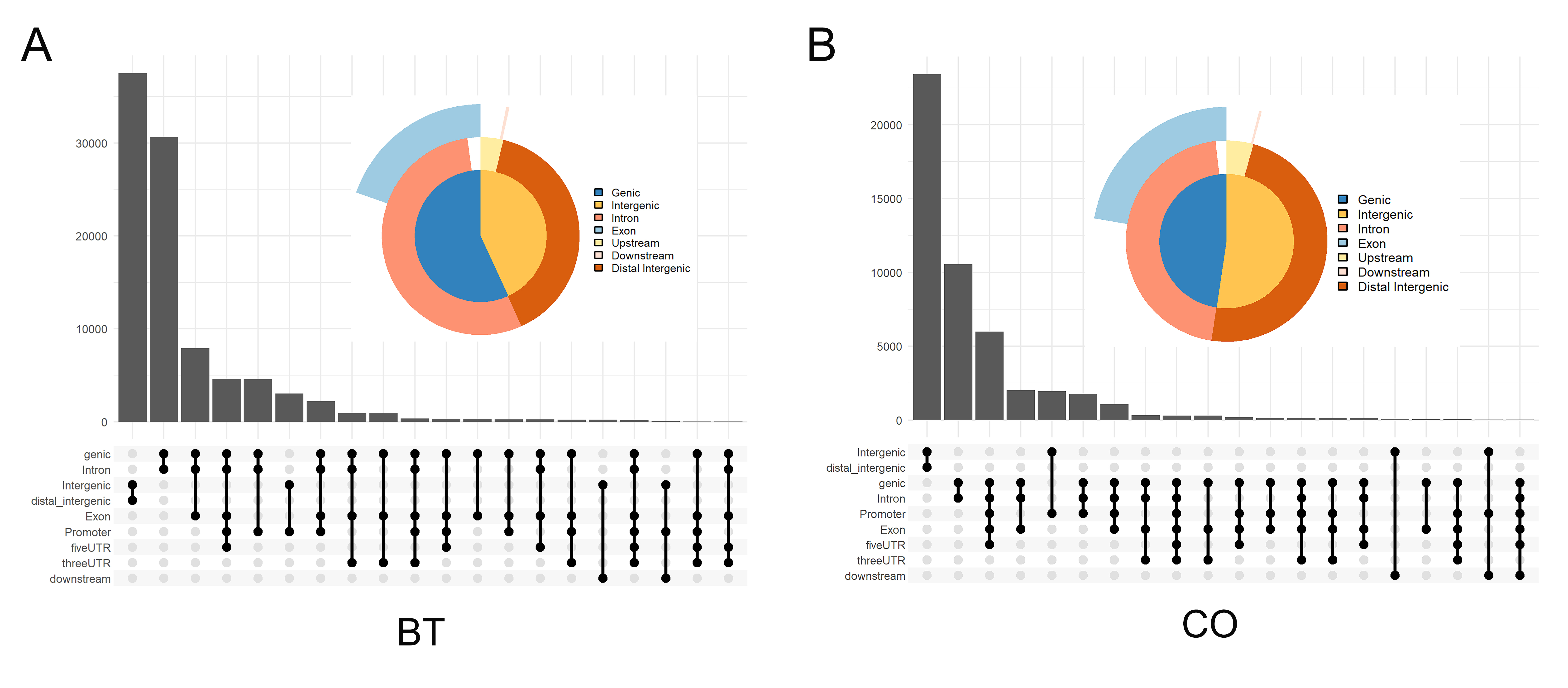

Supplement: Supplementary file 1 [file biomolecules-13-01137-s001.zip › Supplementary Figure/Figure S2.jpg]

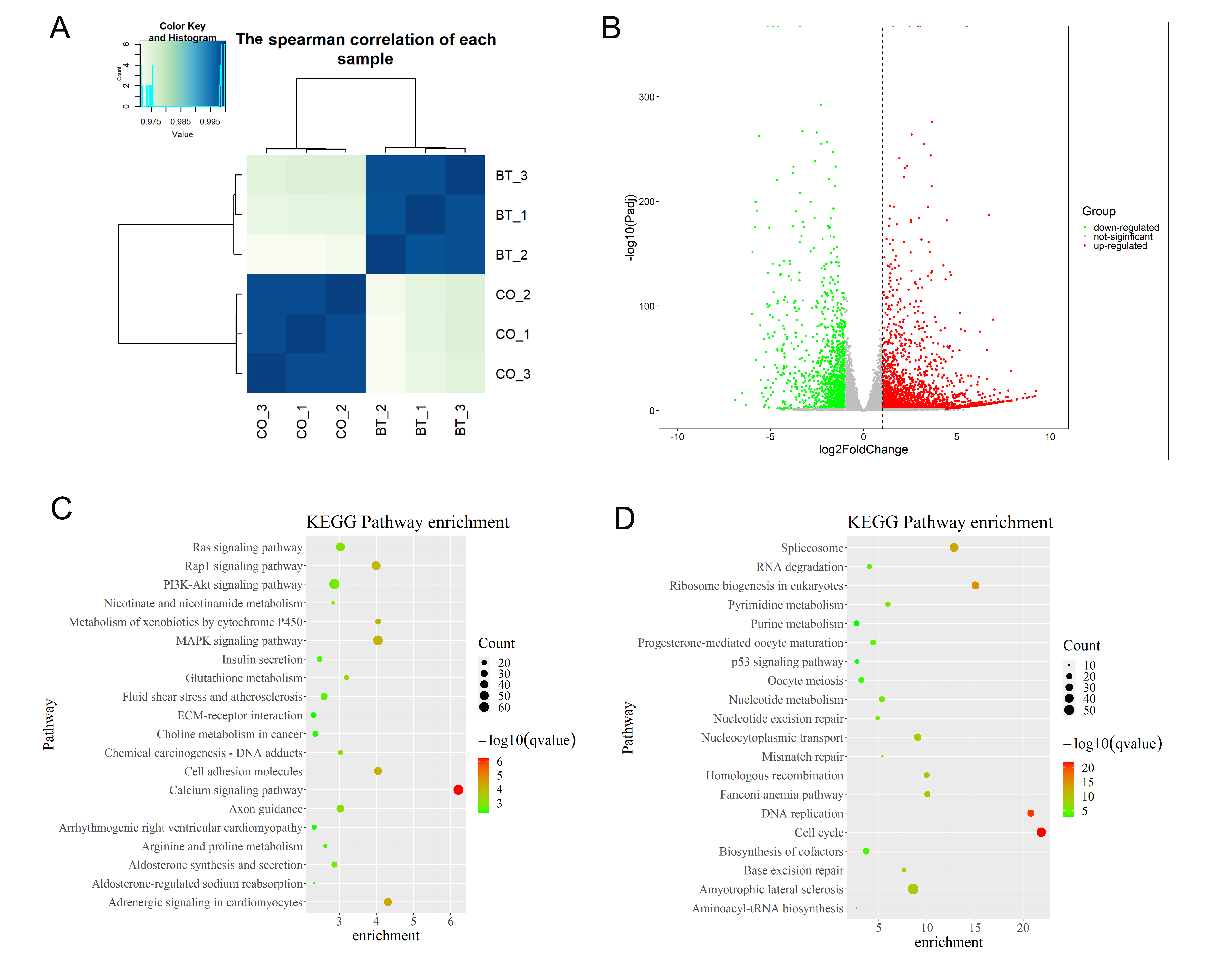

Supplement: Supplementary file 1 [file biomolecules-13-01137-s001.zip › Supplementary Figure/Figure S3.jpg]

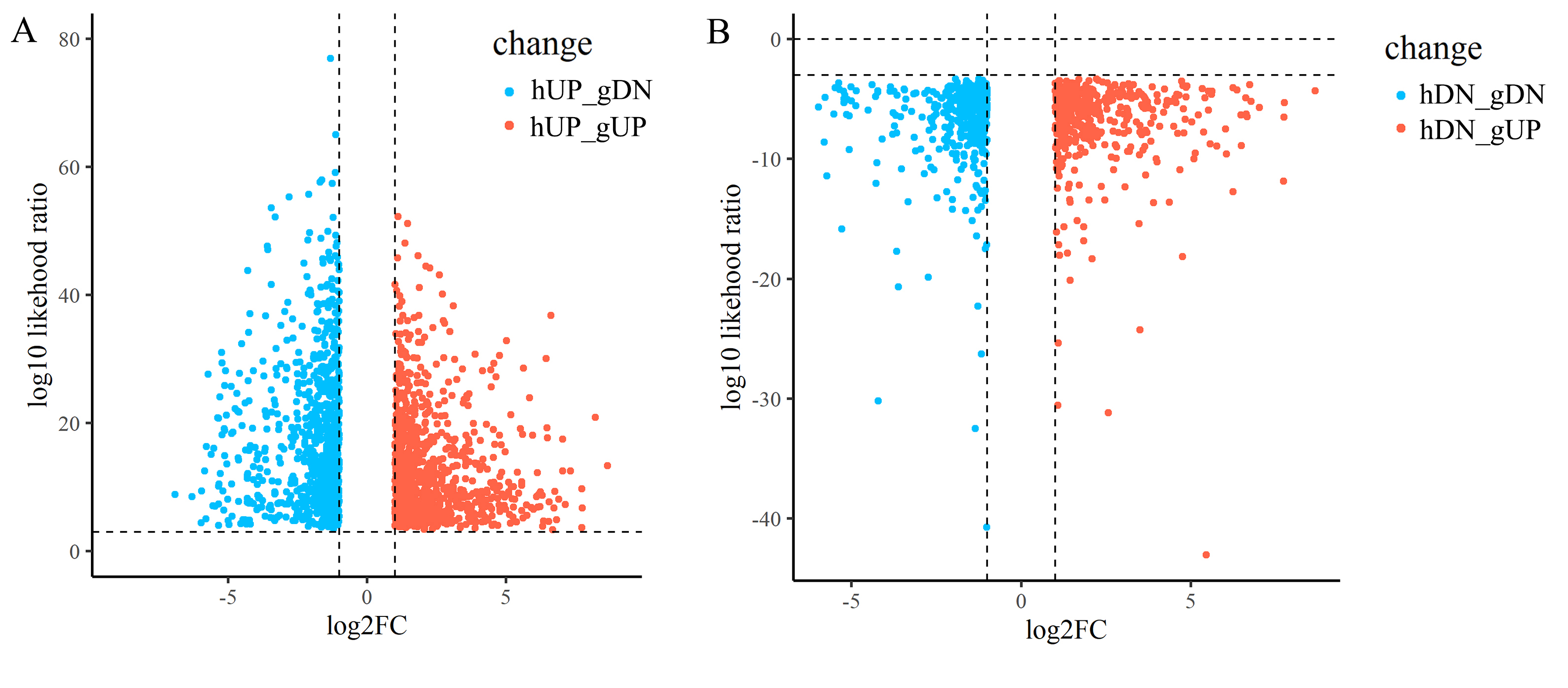

Supplement: Supplementary file 1 [file biomolecules-13-01137-s001.zip › Supplementary Figure/Figure S4.jpg]

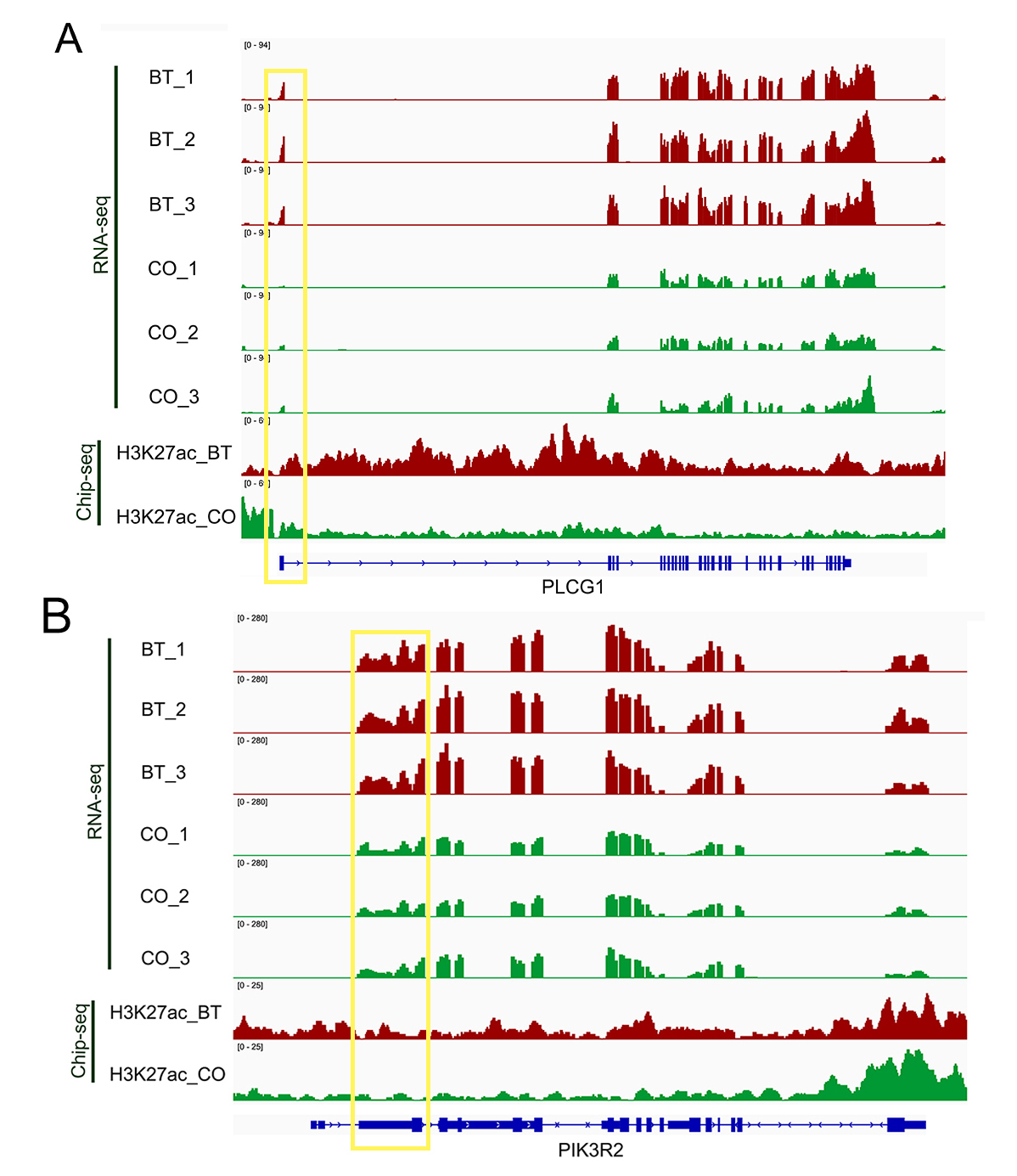

Supplement: Supplementary file 1 [file biomolecules-13-01137-s001.zip › Supplementary Figure/Figure S5.jpg]

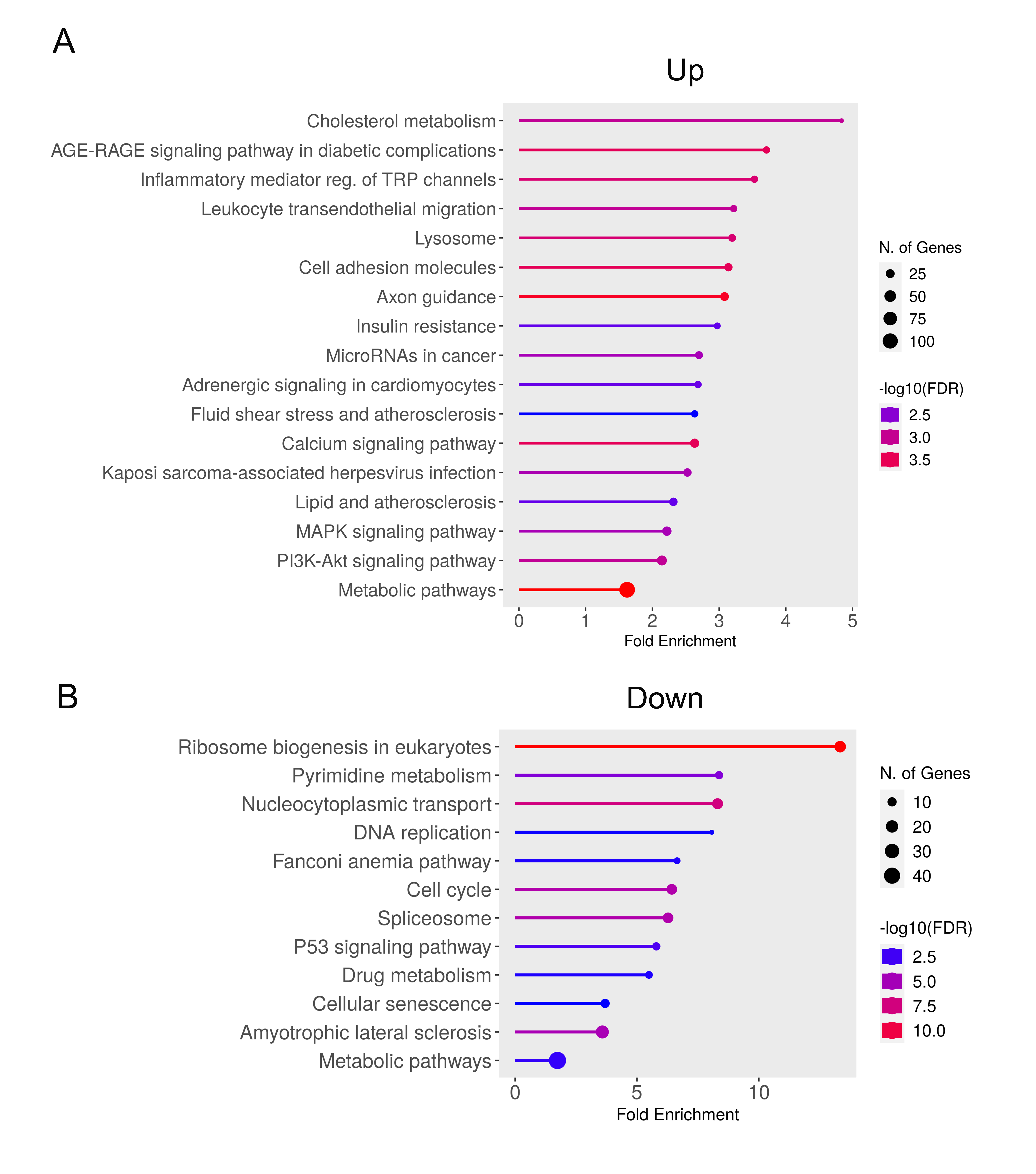

Supplement: Supplementary file 1 [file biomolecules-13-01137-s001.zip › Supplementary Figure/Figure S6.jpg]
